# Us and artificial intelligence: Questioning agency and the need for critical and humanist perspectives

**DOI:** 10.12688/openreseurope.21477.1

**Published:** 2025-10-16

**Authors:** Marco Guglielmo, Petra Ahrweiler, Oscar Barberà, Simon Dario Brockschmidt, Nathan Critch, Ana Luisa de Moraes Azenha, Alejandro Fernández Del Río, Crystal A. Ennis, Pedro García Guijarro, Eva Gomís Jaen, Darcy Luke, Blanca Luque Capellas, Michal Malý, Masoumeh Iran Mansouri, Andrea Medrado, Ben O'Loughlin, D'arcy Ritchie, Mar Sánchez Montell, Carla Sentí Navarro, Saori Shibata, Henry Snowball, Andreu Teruel, Bradley Ward

**Affiliations:** 1 Constitutional Law, Political and Administrative Sciences, Universitat de Valencia, Valencia, Valencian Community, 46010, Spain; 2 Technology & Innovation Sociology / Social Simulation Laboratory (TISSS Lab), Johannes Gutenberg University Mainz Institute of Sociology, Mainz, Rhineland-Palatinate, 55128, Germany; 3 Department of Politics, The University of Manchester, Manchester, England, M13 9PL, UK; 4 Portulans Institute, Portulans Institute, Washington D.C., District of Columbia, 20005, USA; 5 Institute for Aread Studies, Leiden University Institute for Area Studies, Leiden, South Holland, 2300RA, The Netherlands; 6 Institute of Political Science, Charles University, Prague, 15800, Czech Republic; 7 University of Birmingham School of Computer Science, Birmingham, England, B152TT, UK; 8 Department of Communications, Drama and Film, University of Exeter Department of Film Studies, Exeter, England, EX4 4QJ, UK; 9 Department of Politics, International Relations and Philosophy, Royal Holloway University of London Department of Politics and International Relations, Egham, England, TW20 0EX, UK; 10 Department of Poltiical Science and International Studies, University of Birmingham Department of Political Science and International Studies, Birmingham, England, B15 2TT, UK; 11 School of Languages, Arts and Societies, The University of Sheffield Faculty of Arts and Humanities, Sheffield, England, S3 7RA, UK; 12 Department of Politics and International Relations, University of Southampton Politics and International Relations, Southampton, England, SO17 1BJ, UK

**Keywords:** Artificial Intelligence, Critical Theory, Political Agency, Feminism, Decolonial Theory, Political Participation

## Abstract

This essay explores how Artificial Intelligence (AI) is reshaping political and social agency, arguing for the need to ground AI research in critical and humanist perspectives. While AI technologies are increasingly integrated into public infrastructures, their development is often driven by market logics that prioritise efficiency, prediction, and optimisation at the expense of democratic participation, epistemic plurality, and environmental sustainability. The essay results from a two-day international workshop held at the University of Valencia, which brought together interdisciplinary researchers to engage in experiential, reflexive, and collaborative theory-building. Using world cafés, scenario-building, and AI-assisted role-play, participants collectively identified five key challenges of AI-mediated social life: the ideological framing of efficiency, exclusions within participatory processes, the epistemological authority of AI systems, the erasure of historicity, and the instability of predictive infrastructures. In response, we propose a minimal framework to reorient AI research toward feminist, decolonial critical agendas. These include reclaiming predictive power, critically assessing participation and exclusion, and addressing the environmental and geopolitical dimensions of AI. We also reflect on the meaning of research impact in AI scholarship, advocating for a broader conception that embraces critique, friction, and reflexivity. Overall, the essay argues that reclaiming agency in the age of AI requires not only better software-design or regulations but a fundamental rethinking of the social relations, epistemologies, and political imaginaries of technological development.

## Introduction

Artificial Intelligence (AI) is no longer a speculative horizon. It is increasingly embedded in the infrastructures that shape our societies, institutions, and everyday lives. As AI technologies become central to how decisions are made in healthcare, warfare, education, labour markets, or public administration, to name a few areas, they carry with them a set of powerful expectations: greater speed, better outcomes, and more reliable forecasts. But these expectations are not simply technical; they are shaped by a particular set of values and assumptions about how society should function. As the critical theorist of technology Evgeny Morozov (2024) has brilliantly noted, they reflect a broader logic of Panglossian neoliberalism, a view promoted by tech entrepreneurs and platform builders that suggests ‘we already live in the best of all possible worlds’ and that ‘there is no alternative to the market-driven provision of our tech infrastructures’. This essay starts from the premise that Panglossian promise conceals the assumptions through which the implementation of AI in social and political relations is being performed. This is problematic as, under this perspective, AI may contribute to exacerbating social exclusion, hasten war and democratic decline.

AI, broadly conceived as ‘any machine or algorithm that is capable of observing its environment, learning, and based on the knowledge and experience gained, taking intelligent action or proposing decisions’ ( Duberry, 2022: 5), is becoming part of citizens’ social and political relations. As allegedly intelligent machines reshape how institutions operate, how knowledge is validated, and how people interact, they also reconfigure agency: who is able to act, on what, and with what consequences ( List, 2021). Academic attention and public debates on AI have been burgeoning in the last ten years (see: Supriyanto & Saputra, 2022; Ulnicane & Erkkilä, 2023; Zuiderwijk *et al.*, 2021). However, research efforts often remain caught between technical optimism and regulatory caution, without space to reflect on the broader consequences for democratic life, the future of work, social inclusion, or collective imagination of AI-fying social and political relations. By examining recent literature on addressing AI biases through institutional regulation and software design ethics ( Coeckelbergh, 2025; Djeffal *et al.*, 2022), we can also identify key blind spots and seek fresh perspectives. If we affirm that people should have the right to understand and shape the technologies that increasingly govern their lives, then we must ask: who is included in this ‘we’? Whose experiences, values and forms of knowledge count? And who is most at risk of being made invisible, replaceable, or excluded altogether?

This is not just a conceptual problem; it is also a timely one. The hype surrounding Artificial General Intelligence (AGI) has helped reinforce the idea that AI development is on a one-way track, driven by inevitable technical progress. We question this narrative of temporal inevitability and telos, opening paths instead to explore the relationship between technological innovation and contiguous and changing time practices of adaptation and resistance. As Morozov (2024) also argues, the inevitability narrative can serve to distract from more grounded and political questions: ‘If AGI isn’t just around the corner, the debate about the future of this technology becomes more open-ended’, potentially allowing us to question ‘the implications of leaving its development to market forces’. The urgency, therefore, lies in reclaiming a space for critique, for imagination, and for the articulation of alternatives that challenge the inevitability narrative surrounding AI ( Crawford, 2021; McQuillan, 2022). What remains overlooked is a more thorough examination of the social and political relations that underlie the integration of AI systems, the tensions they carry, and the possibilities they foreclose. This essay aims to prompt a theory development process to offer just that. It brings together the reflections, provocations and shared questions that emerged from a two-day international workshop held at the University of Valencia in September 2025. The workshop gathered researchers and practitioners from different disciplines and countries ^1^ to reflect on AI not as a standalone object of study, but as a set of processes entangled with politics, knowledge, and the reproduction of unjust social relations. Through a series of collective sessions, including experiential methods as world cafés, role-plays, and scenario-building exercises, we questioned how AI uses and narratives shape our own positions, assumptions, and research practices.

Rather than offering final answers, this essay develops a minimal framework for critical and humanist perspectives on AI, politics and society. This framework unfolds from identifying key challenges in researching AI in social sciences and humanities, the problematisation of how research can be impactful and, on this ground, which principles should inform alternative agendas and perspectives tackling social exclusion and the risk of agency-displacement to machines. These are the core elements of the essay’s structure. After presenting how we discussed these topics, we propose five core challenges. These include: (1) the dominance of efficiency as a framing device for AI systems; (2) the gap between promises of participation and the persistence of exclusion; (3) the emergence of AI as an epistemological actor; (4) the erasure of historicity and narrative complexity; and (5) the tension between predictive expansion and infrastructural fragility. Each of these challenges is presented not as a siloed theme, but as a way of rethinking what it means to act, to research, and to imagine in AI-mediated societies.

Next, we propose concepts to pluralise and politicise research impact, what it means, who defines it, and how it should be enacted. Drawing from the workshop’s interactive format, including the dialogue with AI-generated political personas, we explore the tensions between stakeholder-driven understandings of impact and more critical, pluralist approaches that emphasise reflexivity, resistance, and ethical discomfort. In doing so, we propose a broader interpretation of *impactful* research, one that includes challenging dominant views and narratives and unsettling settled routines.

Finally, we sketch three priorities for informing open-ended agendas on AI: reclaiming predictive power, rethinking participation and exclusion, and addressing the environmental and geopolitical asymmetries behind AI infrastructures. These are not definitive roadmaps, but minimal conditions for research committed to alternative presents and futures. Altogether, the essay develops the argument that critical and humanist perspectives on AI are not a luxury or an add-on, but a necessary ground for the same possibility of redirecting technological development toward the common good.

## Generativity and reflexivity: Experiential theory development

Understanding the shifting dynamics of political agency in AI-mediated social relations requires novel frameworks and concepts. This was the rationale for developing a collective, grounded, and situated mode of inquiry to prompt fresh perspectives on the relations between AI, society, and politics ( Halsema, 2003). In this section, we briefly present the methods of discussion of the workshop and how they link to the resulting theoretical developments.

Rather than approaching AI as an abstract object of critique, the workshop was conceived as an immersive methodology in itself. The aim was to foster not only interdisciplinary dialogue but also epistemic reflexivity, enabling researchers to explore how our own positions, assumptions, and methods are entangled with the very phenomena we study ( Tarr *et al.*, 2018). Through a series of plenaries and lab sessions, participants collectively interrogated the role of AI in shaping social relations, challenging research practices, and enabling or constraining political agency ( Cornish *et al*., 2023).

### From concept to praxis: Designing for generativity

The event was grounded in a deliberate refusal of pre-packaged disciplinary silos. Instead, we adopted an open-ended and iterative structure, inviting participants to contribute actively to the shaping of the agenda. The initial concept note outlined three transversal perspectives, as entry points to examine how agency circulates between humans and machines: These were the challenges we see as inherent in the proliferation of AI, how we as researchers can productively impact the development of AI, and the need to identify the most pressing agendas for future research. This design served a dual function: it provided enough scaffolding to orient discussion, while simultaneously leaving space for the generativity of unanticipated concerns, ideas, and modes of engagement ( Ball, 2012).

Importantly, the workshop was oriented toward experiential collaboration. The 25 participants were invited to co-produce situated knowledge. Instead of submitting papers, participants completed a pre-workshop open-ended survey. This survey asked them to propose the challenges they considered in researching AI and agency, the potential impacts of such research, which agendas are more urgent on this broad topic, and their expectations about how the workshops would contribute to these ideas and goals.

The organisational logic reflected an ethos of horizontal learning ( Freire, 1994). Early-career researchers were not only welcomed but structurally supported, ensuring that power asymmetries did not reproduce within the space of the workshop: all those with more senior or wealthier job positions agreed not to receive participation awards to distribute support to early career and precarious researchers. While the participants reflected disciplinary, gender and geographical diversity, a limitation of the workshop is that all academics are based in institutions in the Global North. This limitation was discussed during the workshop, which formed the basis for designing further activities.

In what follows, we briefly present the workshop structure. On day one, participants came to know each other starting from their own experiences and aims, and yet practised collaborative methods for theory development. On day two, we drew on the previous discussions to devise the principles for fresh research agendas on AI, politics and society.

### Method as encounter: World-Café and role-play

The opening plenary, ‘Researching GenAI: Challenges’, began with short interventions from participants, followed by a clustering of shared dilemmas. These ranged from concerns about algorithmic epistemologies and exclusionary infrastructures to questions of resistance, regulation, and the political imaginaries that underpin AI adoption. Importantly, the plenary was not designed to resolve these issues but to surface tensions and provide conceptual material for the collaborative lab to follow.

In the first laboratory session, participants engaged in a World-Café format structured around the challenges identified earlier ( Löhr *et al.*, 2020). The participants were randomly assigned to three tables with prompts inferred from the plenary session (‘efficiency, or what?’, ‘who can(not) participate?’, and ‘how do we know what AI knows?’) plus a fourth table with no pre-defined themes. Moving between discussion tables every twenty minutes, each anchored by a host who retained and synthesised earlier conversations, participants cycled through multiple perspectives. This approach added layers of complexity to the initial prompts with each round, enabling us to identify areas of disagreement and open debates.

The emphasis on experiential methods was further expanded in the second lab-based session, which followed a collective discussion on the theme ‘Whose Impact and What For?’. The discussion addressed whether and how researchers can bring positive impacts when interrogating AI, society, and politics. Here, we adopted a role-play method to situate ourselves in the position of humans interacting with quasi-agentic AI chatbots embodying distinct political actors. These included a fictionalised Prime Minister, a conservative editor-in-chief, a community organiser from the Amazon region combating deforestation, and a high-ranking EU bureaucrat. Also in this session, there was a fifth table where participants could design their own agent. Participants engaged in role-play, utilising the agent designed in ChatGPT 4-o to assume the role of researchers seeking to open dialogue with these figures and explore pathways for impactful research.

### Reflexivity as orientation

Throughout the workshop, reflexivity was not an add-on but a constitutive principle ( Farrugia, 2013). Participants were not only asked to reflect on AI as a social and political phenomenon, but on their own positionalities, disciplinary assumptions, and methodological commitments. This reflexivity was at the core of the final laboratory session, which encouraged participants to devise shared agendas, thematic synergies, and overarching principles for the future of research on AI, politics and society. These principles informed the commitment to follow up with further networking activities, primarily focused on prompting humanist and critical perspectives on AI. This essay represents one such collective outcome, an attempt to articulate critical and humanist perspectives on AI grounded in shared experience and iterative reflection.

Ultimately, the methodology adopted was not merely a means of organising a workshop, but a practice of epistemic care attuned to the risks of abstraction, hierarchy, and closure that often accompany AI discourse. By foregrounding generativity and reflexivity as methodological principles, the workshop sought to reclaim agency, not just analytically, but in how we organise our knowledge practices, build our research communities, and imagine our futures with (or without) AI. This is crucial not to replicate the same data extractivist logics we critique in tech industries ( Lehuedé, 2024).

In what follows, we articulate how these collective reflections about the challenges and impacts of research started developing key concepts for critical and theoretical, much-needed approaches to AI, society, and politics.

## Us and AI: Challenges

Artificial Intelligence is often heralded as a technological breakthrough that promises to reshape society for the better, delivering faster services, more efficient governance and administration, and enhanced decision-making ( Duberry, 2022; Dunleavy & Margetts, 2023). The promise of AI is not only that societal goals can be achieved faster and better. Techno-entrepreneurs and politicians emphasise how everything will also be cheaper, often obscuring how these alleged financial savings come with enormous costs for natural resources ( Crawford, 2021) and huge human exploitation ( Muldoon *et al*., 2024). In this discourse AI offers to improve innovation itself, since all possibilities can be generated and considered immediately, so “we” can invent *more*, *better*. These framings are not ‘out there’ by chance. The promise of the Panglossian world, always almost-there-to-come (see Introduction) is a purposeful ideological construction seeking to obscure the deeper tensions about the role of AI in society ( Gilbert & Williams, 2022). This section brings together the reflections and provocations that emerged from the workshop to propose the core of a critical theorisation of the challenges AI poses to social and political relations.

Through an exercise of grounded and reflexive theory development, the participants came to set the contours of key epistemological and societal challenges of AI-fied societies. Ahead of the workshop, participants were invited to share their perspectives on the most urgent challenges posed by AI. Three main themes emerged from these reflections: first, the ‘problem’ of how to conceptualise alternatives to AI as efficiency-maker, especially given increasing evidence of the flaw of this promise ( Bracci, 2022; Henman, 2020); second, the tension between envisioning AI-assisted or driven participatory processes to reform declining democratic systems ( Sieber *et al.*, 2025) and the a-democratic-by-design attributes of most AI systems ( Chehoudi, 2025); third, the epistemological challenge of uncovering the promise of AI as the leader of a new data-driven wave of political and social relations ( Coeckelbergh, 2025) and how this clashed with evidence of the ongoing exclusion of multiple human perspectives ( Noble, 2018). As the generation of key concepts saw the rotation of participants around discussion tables, during the laboratory session, some discussants identified two further related challenges concerning, respectively: who masters time and temporality in human-AI relations and the clash between the transfer of predictive power to AI machines ( Coeckelbergh, 2021) and the unpredictability of whether the whole AI infrastructure may collapse because of its own premises and promises ( Peterson, 2025).

### Efficiency. Or what?

The first core challenge concerns the powerful imaginary of AI as a driver of efficiency, a trope that pervades public discourse, government narratives ( Fourcade, 2016), and corporate promises ( Amoore *et al.*, 2024; Hirsch-Kreinsen, 2024). Whether in national economies, policymaking, service delivery, research practice, or even deliberative democratic processes, AI is increasingly framed as a solution to longstanding performance gaps ( Zuiderwijk *et al.*, 2021). However, as the laboratory discussions made clear, the assumed link between AI and improved efficiency is highly contingent and ideologically charged. Think about AI-driven facial recognition in warfare and its implementation in drones ( De Swarte *et al.*, 2019): the promise of making warfare more efficient, that is, targeting individuals with ever greater precision, comes with the idea of more effective and less costly military operations, with less collateral damage to civilians ( Sauer & Schörnig, 2012). In a different area, an AI-outsourced software can quickly manage citizens’ demands for public health services ( Khosravi *et al.*, 2024). Given a set of parameters, the AI can allocate services to deserving citizens more efficiently than a human bureaucrat: the promise here is to improve the fulfilment of an essential right, even when the AI rejects a demand. Yet, as diverse as they are, these cases unveil already-present dystopias masked as the promised land of efficiency. Are these the presents that, as researchers, we want to contribute to? And how can we contribute to alternative models of technological integration into social relations?

Participants drew attention to the scale-specific impacts of AI. At the individual level, there is a strong gravitational force attracting us to use AI tools to assist with tasks such as summarising texts or scheduling activities. This is particularly true in intellectual jobs under neoliberal regimes, whereby there is high pressure to perform multiple functions with heavy cognitive loads ( Vercellone, 2014). At the organisational level, particularly in the private sector, the empirical evidence of productivity gains remains mixed ( Bankins *et al.*, 2024). At the state level, where governments are increasingly investing in AI to compensate for depleted capacity, the picture is equally ambiguous ( Neumann *et al.*, 2024). Furthermore, while AI might accelerate the processing of a service request, the quality and equity of the outcome remain shaped by structural conditions, be they institutional, socio-economic, or legal.

The collective reflections problematising this gravitational yet ideological attraction of AI as an efficiency-booster have led to a process of generative deconstruction of the hidden problems of efficiency: when quantified as the process of generating more output at minor cost at the most enormous scale and standardised shape, efficiency does not equate and more likely contrasts with creativity and democratic innovation ( Edwards, 2001). When creativity and diversity are overlooked as a result of the reproduction of established power relations and/or for their innate non-quantifiable essence ( Moore, 2017), social complexity and lived experiences are cast as inefficiencies to be minimised. As one participant put it, the problem lies not only in what AI can or cannot do, but in what alternative values get displaced when efficiency becomes the overriding goal.

The deconstruction of efficiency was ground to envision alternative imaginaries, rooted not in making everything faster or cheaper, but in transforming the goals of public and private systems. Creativity, lived experiences, ecological responsibility, and collective well-being, none of which lend themselves to quantification, are the marginalised principles that can form the base of alternative human-to-technology relations. Transformative visions of AI prioritise happiness, relationality, and planetary care over sheer optimisation. While it is not the primary function of the research community to set alternative agendas, researchers committed to alternative social relations can play a crucial humanist and critical role in unmasking the deleterious effects of quantified efficiency and providing ground for alternative use of human-centred technologies.

### Who can(not) participate?

While the first challenge has to do with the ideological concepts and modes of operation of AI, the second challenge arises from the widespread assumption that AI can enhance participation, be it in democratic governance, public service co-design, or civic engagement ( König & Wenzelburger, 2020). While this promise is often invoked by policymakers and developers alike, the workshop surfaced multiple ways in which AI-driven systems within unjust social relations may, in fact, exacerbate exclusion.

The starting point is to think about participation, in what? As data-hungry systems, AI requires all citizens to participate in feeding the machine ( Muldoon *et al.*, 2024), be it by conceding personal information or in helping recognise images and languages. In the white West, this is proposed as an equal exchange for receiving ‘free’ digital services and spaces of socialisation ( Jordan, 2015). On the other hand, the hidden labour of AI, digital platforms, and the building gigantic databases are displaced on and sourced from the exploited labour of women, migrants, and digital workers in the ‘Global South’ ( Altenried, 2020; Altenried, 2022; Fuchs, 2018).

This premise is critically damaging to the same possibility of alternative AI systems. Forced data input is antithetical to the co-creation of AI. Acknowledging these striking dialectics requires taking into consideration that participation is always mediated by systemic power and its effects on access, literacy and representation in digital and offline spaces ( Fuchs, 2019). AI systems are often built on infrastructures and data sets that reflect dominant interests, and as such, they risk encoding social inequalities into their outputs ( Mühlhoff, 2025). As one participant warned, citizens' agency in AI governance is frequently assumed rather than supported. In practice, structural exclusion by class, gender, race, age, access to education and health, geography, and disability remains pervasive.

The deployment of AI in existing participatory processes does not necessarily resolve this problem. Instead, it may deepen it by shifting the terms of engagement from human deliberation to machine filtering and optimisation, which can limit the opportunities for true communication and collaboration, prioritizing efficiency above diversity. If AI is used to ‘streamline’ citizen input or ‘enhance’ participatory budgeting, who decides which inputs matter? Who interprets dissent? Who gets filtered out? The use of AI can also compromise transparency and accountability, as complex algorithms can operate as “black boxes” with decision-making criteria that are difficult for participants to access or understand. Lastly, the collection and processing of personal data raises ethical considerations around control and consent as well as data privacy and surveillance.

Answers to these questions define a multifaceted space for alternatives, ranging broadly speaking from envisioning molecular changes and experimentations in AI-humanistic models to the need to resist AI altogether as inevitably ingrained in structural domination. One example of the first position is the vision of AI agents as ‘rights mediators’ assisting citizens in navigating state labyrinthine bureaucracies ( Floridi, 2020). On the other hand, thinking about the problem of AI in workplace governance, where digitalisation intersects with gendered and racialised forms of surveillance, participation should be considered in its most material, embodied sense as anti-AI control of bodies and experiences ( Woodcock, 2021).

However, ‘Participatory AI‘ that has a seriously critical and emancipatory stance is not completely out of the horizon ( Birhane *et al*., 2022): In a time where it might not be feasible to forbid AI as a technology, it is worth thinking of a radically different approach to inclusive technology co-design that can replace existing systems combining more legitimacy and social responsiveness with competitive performance. This was, for example, done by the international research project AI FORA ( Ahrweiler, 2025) which explored, within a culturally diverse set of case studies across the globe, whether and how participatory modelling in multi-stakeholder settings of the ‘Quadruple Helix’ ( McAdam & Debackere, 2018) including vulnerable groups can support the context-sensitive design of ‘Better AI’ in welfare systems.

Current algorithmic tools and administrative data used in welfare assessments often do not reflect cultural values and discourse but reflect politically unintended bias and reinforce existing structural inequalities ( Köbis *et al*., 2022; Mitchell *et al*., 2021; Pagano *et al*., 2023); without careful co-design and continuous evaluation, AI risks institutionalising bias rather than correcting it. In AI FORA, a process was developed around a dedicated modelling environment to effectively capture the complexity of welfare systems, reveal hidden decision-making dynamics, and serve as a testbed for evaluating alternative policies and AI rule sets ( Ahrweiler *et al*., 2024). Models allowed stakeholders, including vulnerable groups, to experiment safely with new rules and explore their systemic consequences before real-world deployment. Gamification and serious games facilitated stakeholder deliberation around fairness, trade-offs, and algorithmic design; this combination enhanced stakeholder understanding of algorithmic systems and encouraged collective reflection. Deliberations about the characteristics and dynamics of existing systems led to requirements for desired system futures that implied “changing the algorithm”. Training data for new systems was produced by simulations using the changed algorithm to check on system performance at population level. Participatory approaches prominently included both, social service frontline workers and policymakers, to translate modelling results into policies and practice ( Ahrweiler & Gilbert, forthcoming). Results showed that participatory modelling approaches, particularly when combining agent-based modelling and gamification, can lead to more legitimate, context-sensitive, and equitable AI systems for public social services.

The critical question is therefore not whether AI can enable participation, but whose participation is enabled, under what terms, and at what cost. Democratising AI requires more than adding citizen inputs to pre-trained models; it entails re-designing the architectures of inclusion and re-politicising the values embedded in AI governance. Crucially, the generative reflections from participants in the workshops became sites of mutual learning. Citizens-centred AI perspectives can provide examples of resistance through platforms to resistance-based approaches, and vice versa. Feminist perspectives in labour are critical not to overlook the areas of substantial exclusions from formally open participatory processes.

### How do we know what AI knows?

A third challenge centres on AI as a producer of knowledge, and the implications this holds for epistemic authority ( List, 2021). In the workshop, participants probed the consequences of AI systems not only processing data but generating representations of the world, which increasingly function as accepted truths in forging meaningful interpretation of events and decision-making processes ( Carabantes, 2020). Understanding AI as epistemological machines shaping, if not chaining, how we know and interpret phenomena is the necessary starting point of critical and humanist perspectives.

To begin with, the dominant notion of knowledge in AI-driven societies must be deconstructed. Knowledge is not a neutral accumulation of quantifiable data to be assembled in fact representations ( D'Ignazio & Klein, 2020). Rather, knowledge is itself a social process, embedded in power relations, partial perspectives, and normative assumptions. In contrast, AI-epistemological machines tend to reify data, presenting probabilistic outputs as objective insights ( Micheli *et al.*, 2020). The evidence of the perils of such assumptions is already clear when AI-biased knowledge production is used in contexts such as criminal justice and welfare distribution, with dramatic effects on underrepresented and marginalised communities ( Gillespie, 2024; Phan & Wark, 2021).

A related concern focuses on the ‘who’ of knowledge-production. AI platforms, especially generative systems, can draft biographies, policy suggestions, and even legislative drafts, but without accountability, reflexivity, or contextual understanding. A data-driven ‘picture of reality’ may reflect statistical correlations but lacks the experiential depth or political contestation through which democratic knowledge is constructed: these are all processes of knowledge (de-)construction, exchange and critique that cannot exclude human reflexivity and agency.

Moreover, a bias-loop problem raises the key issue of the potential enclosure of contestation. AI systems are trained on data normalising existing forms of exclusion and domination, which do not contrast with platform companies' protocols ( Danaher, 2016); without processes of re-structuration, and the inclusion-by-design and infrastructures of marginalised voices and experiences, AI enclose humans in a-spatial loops. Further, as the actual AI infrastructure is heavily North-West centric, the promise of data-driven politics for the common good is hampered by an inherent re-colonialising tendency ( Hepp *et al.*, 2022). As one participant put it, we risk entering a regime where ‘AI creates knowledge, AI judges knowledge, and AI reproduces knowledge’, a closed epistemic loop with limited space for human dissent or pluralist validation.

This scenario raises critical questions: what counts as legitimate knowledge in AI societies? How do we retain the capacity to interrogate what AI tells us? And how do we resist the deskilling of human judgment, especially when productivity pressures incentivise deference to machine output?

The core concept driving critical perspectives is therefore the constant reclaiming of humanism, not as a nostalgic return to pre-digital modes of knowing, but as an insistence on situatedness, plurality, and narrative agency ( bell hooks, 2014). The challenge is not only to critique what AI knows, but to reassert how and why we know what AI knows. Reflecting the challenge of participation, the nucleus of humanism shapes a debate with different positions that need to construct bridges of epistemic solidarity. On the one hand, there is the idea that alternative uses of existing AI systems can improve the process of knowledge production through constant and coordinated injection of situated, non-dominant data, discourse and perspectives. On the other hand, there is the position claiming the right to disconnect from the machine ( Kuntsman & Miyake, 2022) and to re-assert the primacy of humans in co-creating knowledge from and about social processes.

### Human historicity and a-temporal machines

In relation to the problem of knowledge, there is the challenge of how to identify what is lost when AI becomes central to how we imagine social issues and solutions. While being heavily territorialised in the so-called Global North and imposing the fast pace of quantified productivity, AI systems are marked by an absence of historicity, by their incapacity to grasp or represent temporal depth, political contestation, or narrative complexity ( Coeckelbergh, 2022).

Several examples illustrated this point. Generative AI systems can assist in drafting policy or proposing interventions, but they are poorly equipped to understand how problems evolve over time, how they are shaped by struggles, or how they reflect unresolved injustices. One participant captured this vividly: ‘GenAI is not a Deus ex machina, yet most people expect it to be’. This expectation not only misrepresents what AI can do; it distorts how humans approach problems, encouraging shortcut solutions and flattening the cognitive labour of critical diagnosis ( Berry, 2014).

This challenge is compounded by what a participant called ‘a need for narrative literacy’. To be ‘AI literate’ is not simply to understand how models work, but to comprehend how they organise sequences of meaning, how they produce stories, select facts, and filter relevance. Without such literacy, there is a danger that AI-generated representations displace human storytelling, with profound implications for education, memory, and identity.

The epistemic flattening produced by GenAI echoes broader cultural patterns of amnesia, acceleration, and depoliticisation ( Burnham, 2017). When AI becomes the primary source of explanation or interpretation, we risk displacing the slow, contested work of political narration, where people make sense of their lives, communities, and aspirations. In this sense, historicity and narrative literacy are not technical add-ons, but core conditions for democratic agency.

### Between predictive super-power and un-predictable collapse

The fifth challenge interrogates a central assumption behind the current AI boom: that its infrastructures are robust, expanding, and inevitable, primarily as they take over predictive power ( Mühlhoff, 2025). If humans delegate prediction to the machines, they are chaining themselves to the need for more and more powerful calculators and endless computing capacity. Yet participants urged a more critical stance, recognising the fragility and unsustainability of the models underpinning AI. In other words, we may be conceding predicting power to technological systems which are in many ways unsustainable, prone to crises and collapses which we are not ready for.

This tension and paradox is well explained by the environmental costs of AI development. On the one hand, the extractive attribute of AI is well known: energy consumption, material extraction, and labour exploitation, often outsourced to the ‘Global South’, has been studied at large ( boyd & Crawford, 2012; Pirina, 2022). However, this inherent attribute is often presented in political and public discourse as a negative externality, a fissure to be fixed. This material and ideological process serves to make the visible invisible. And indeed, these extractive dimensions are rarely factored into policy discussions while being integral to the global political economy of AI ( Dauvergne, 2022).

The call for exposing what is unsustainable in current AI developments leads to the need for reflections on a seemingly provocative question: what if the business models sustaining AI development are unsustainable? What if the current trajectory mirrors the dot.com bubble, driven by hype, inflated valuations, and faith in endless expansion ( Floridi, 2024)? While these evolutions are over-determined, model collapse cannot be excluded, even less in social research on AI: there is the possibility that AI systems will eventually consume their own outputs, degrading accuracy and eroding creativity. This ‘cannibalisation’ effect, where models recycle their own data until generative saturation, could result in a profound epistemic and economic breakdown ( Fraser, 2023). The broader claim is that AI operates within a post-Schumpeterian moment, one where innovation has stalled, monopolies dominate, and competition no longer drives novelty. In this context, the predictive power of AI is not a neutral tool, but an ideological force, reinforcing a linear, technocratic vision of the future while foreclosing alternatives.

As with previous challenges, the generative discussion led participants to claim the need for community-based, localised alternatives to the dominant AI paradigm, technologies that serve real needs rather than speculative futures. But to make such alternatives viable, we must first challenge the hegemony of predictive reason and reclaim the possibility of rupture.

## AI, politics and society: Can we make an impact?

The question of research impact, what it means, for whom it is intended, and how it should be achieved ( Bornmann, 2013) was at the core of the second bloc of discussion on the relationship between AI, society, and politics. Rather than adopting a narrow or instrumental understanding of impact, the session sought to pluralise and politicise the term. This exercise was relevant for reframing impact as a spectrum of possibilities, rather than a quantified measurement of research contribution on social and organisational indicators ( Wigginton & Lafrance, 2019).

We began with a collective reflection on the relational infrastructures that researchers are expected to use to generate impact. Institutions, ranging from public administrations to media regulators and civil society organisations, often articulate specific expectations about what research can or should deliver: evidence-based policy recommendations, accurate datasets to address societal challenges, or metrics to evaluate risks and opportunities of emerging technologies ( Bergold & Thomas, 2012). From this perspective, impact is defined mainly by the demands of stakeholders as interventions at specific points, for instance, the delivery or assessment of a policy cycle ( Luchetta, 2012). Starting from this reflection, some participants proposed how research on AI and the co-creation of alternative AI can enhance the role of academics while boosting liberal democratic participation. One example is that of citizen-centred AI tools that enhance rights navigation across EU platforms, to participatory consultations such as the Democracy Shield. These initiatives respond to real and pressing needs for inclusivity, explainability, and regulatory accountability ( Novelli & Sandri, 2025). They illustrate how AI research can be mobilised to bridge civic gaps and facilitate more responsive institutions. However, these examples also point to the constraints of stakeholder-driven models of impact: they often leave little room to question the premises or direction of the institutional frameworks they serve.

This tension led to a broader critical discussion on the need to re-centre humanism and pluralism within the very conception of impactful research. Instead of viewing research as a transactional exchange, delivering answers to pre-defined institutional questions, the session foregrounded a mode of inquiry in which scholars act as facilitators of reflexivity, contestation, and democratic imagination. Such a shift involves a spectrum of researchers’ positionalities in search of areas of dialogue: on the one hand, scholars can contribute to more inclusive policy-making processes, for example, by co-designing participatory mechanisms to include the voices and experiences typically excluded in techno-legal deliberations. On the other hand, there is the need to gain space for research which adopts an adversarial or critical posture towards institutions and organisations, questioning whether existing institutional logics are compatible with democratic goals in the age of AI ( Mansouri & Bailey, 2025).

Indeed, a recurrent theme in the conversation was that critique is not antithetical to impact but a necessary yet dismissed part of the relation between academia, organisations and society. While often tolerated as a niche or dissident perspective, critical research is rarely recognised as impactful. Yet, in fields like AI, where epistemic, normative and infrastructural transformations unfold rapidly and asymmetrically, critique is indispensable ( Lindgren, 2023). It makes visible the blind spots of dominant approaches, resists the flattening of alternatives, and insists on the ethical-political stakes of technological design and deployment. In this light, impactful research is not only that which delivers outputs aligned with stakeholder needs, but also that which disrupts dominant narratives, introduces friction, and reclaims the space for alternative futures. The challenge, then, is not to choose between these two understandings of impact, improved accountability versus critique, but to articulate their tensions to improve their reciprocal spaces of possibilities in moving social relations towards the common good.

To explore these tensions in a situated and experiential manner, the laboratory session invited participants to engage in a simulation exercise: a role-play with agentic AI chatbots, designed with GPT-4o, allegedly simulating different political and institutional actors. Participants were organised into small groups and invited to conduct a dialogue with these synthetic interlocutors as if they were real-life counterparts, prime ministers, editors-in-chief, EU cabinet chiefs, or community organisers (see above). The objective was not to assess the technical performance of the chatbots, but to use them as a medium for reflecting on what it means to do impactful research with, through, or against AI systems.

This format offered a unique vantage point to examine how GenAI systems perform political speech, reproduce normative assumptions, or generate affective responses. In some instances, the exchanges produced moments of surprise: participants found themselves persuaded, provoked, or disoriented by the chatbot’s replies. These moments offered critical insight into the simulation of political agency by GenAI models, how such systems are trained to emulate rhetorical strategies, project ideological stances, or manage dissent. At the same time, the exercise exposed the limits of such interactions. In cases where participants engaged the chatbot posing as a head of government, responses often remained superficial or evasive, failing to sustain substantive discussion on regulatory frameworks or democratic safeguards.

Importantly, the activity did not aim to reach definitive conclusions on the feasibility of AI-simulated political engagement. Instead, it served as a laboratory for reflexive discomfort, a space where participants could experience the contradictions of AI research impact in real time. One particularly illustrative moment emerged when a group of participants, engaging with a chatbot representing a grassroots environmental activist, questioned the ecological footprint of GenAI itself. The agent expressed a form of systemic pessimism, while its very response was being generated through energy-intensive computation. The irony was not lost on the participants, who chose to end the conversation early as an ethical gesture. Such moments underscore the importance of reflecting on the conditions of experimentation when designing interactive research settings integrating AI systems.

Another table, composed of feminist scholars, engaged the chatbot playing the role of an EU foreign policy official. Their questioning focused on the integration of gender perspectives into security strategies, a line of critique often marginalised in traditional policy impact frameworks. While the responses from the chatbot were measured and respectful, they nonetheless surfaced a latent tension: AI systems, even when adopting politically progressive scripts, can re-inscribe hierarchies of knowledge and authority. In this sense, the activity became a site for rehearsing dissent, where participants could test the elasticity of synthetic discourse and examine the boundaries of critique under conditions of simulation.

In sum, the session offered a multi-layered exploration of impact, developing further the need for a pluralistic conceptualisation of the process of making a difference from research to social systems. It created a space of dialectics and agonism between the more pragmatic and policy-driven demands of institutional actors and the epistemic and ethical responsibilities of critical scholars. It used the affordances and constraints of GenAI not to propose ready-made solutions, but to stage questions, surface contradictions, and provoke reflection. In doing so, it affirmed that impact is not merely a matter of external validation or stakeholder alignment, but a process in need of critique and reflexivity.

## Open-ended critical and humanist agendas on AI and social relations

The critical reflections on the challenges and the problematisation of impact have provided valuable orientations to assess the evolving role of AI in society and politics. On this ground, we aimed to develop key concepts and perspectives to devise the minimal foundations for broader agendas on AI, society and politics. Through our collective reflections, we identified three core priorities for varied intersecting areas of investigation: reclaiming predictive power, redefining participation and exclusion, and addressing environmental and developmental asymmetries.

To begin with, there is a need for agendas that reclaim and redirect predictive power ( Nowotny, 2021). To delve deeper into this priority, we started by reflecting on how AI is already reshaping the knowledge infrastructure of academia itself, from how we collect data to how we teach, publish, and disseminate research ( Grossmann *et al.,* 2023). The question ‘what will conducting research in 2035 look like?’ was not posed rhetorically. Instead, it served as an anchor for scenario-building exercises aimed at unpacking the institutional, epistemic and technological shifts currently underway. The purpose of such future work is not to offer deterministic forecasts but to clarify the conditions under which particular trajectories may become dominant, and to imagine interventions that might disrupt or redirect them ( van Witteloostuijn *et al.*, 2022). Defining scenarios and the conditions for them to unfold is one way to advance critical reflections on the alternatives and on the perspectives that are still underexplored. Drawing from participant interests, we took the question of ‘what is the AI doing to labour relations?’ as a perspective to illuminate broader changes. We are becoming accustomed to the use of AI in recruitment, human resources management, and surveillance processes ( Cant, 2020). This happens in the background of discourse and imaginaries, promising work to become more innovative and more productive ( Al Naqbi *et al.*, 2024). What are the possible scenarios unfolding from the dialectics between the promise of productivity and the practices of authoritarian surveillance? And is there any space for alternative uses of AI models in favour of labourers’ creativity? These questions illuminate the potentiality of shifting the perspective of social inquiry. As proposed throughout this essay, to take feminist and decolonial epistemologies ( Stingl, 2015) seriously means shifting how we look at phenomena to shed light on what practices of resistance to and through algorithms ( Bonini & Trere, 2024) have been emerging and forging solidarity bonds that can inform the background against which implementations of AI are assessed ( D'Ignazio & Klein, 2020).

The second priority for novel research agendas relates to the challenge of who participates with AI, and to what end? Today’s AI development is still expert-driven, ‘characterised currently as technically-focused, representationally imbalanced, and non-participatory’ ( Birhane *et al*., 2022: 1): that is to say, at the very least a-democratic. Though Birhane *et al*. report recent participatory methods in the lifecycle of machine learning including auditing, public consultation such as citizen juries, joint problem formulation, model cards and datasheets for information disclosures, and even artefact co-development, they voice a suspicion that these methods might merely serve to aid in the refinement of systems from the viewpoint of industry. Specific pitfalls in participatory approaches to AI innovation need awareness to be avoided. Currently, the composition of stakeholders and the degree of influence they have vary very much between initiatives.

The plea goes for participation that ensures empowerment and equity of participants with wider humanitarian or societal benefits. Participatory AI approaches need to specify the reasons for more stakeholder involvement ( Donia & Shaw, 2021; Iason, 2022) presenting frameworks, best practice recommendations and guidelines on how to do it ( Lee *et al*., 2019; Mohamed *et al*., 2020). Goals, duration, and limitations of the participatory exercise should be clear for participants at all stages of the process. Birhane *et al*. ask for standards for participatory approaches in AI that include claims for reciprocity, reflexivity, and empowerment. As the example of the AI FORA project above shows, to develop a critical and emancipatory Participatory AI approach is complex, research-intensive, costly and needs to be highly diversified to support adaptive local solutions. Tailored methods for stakeholder involvement, anticipation and foresight such as participatory modelling and simulations have to co-evolve alongside. In the end, policy impact depends on more than technical insight: It requires institutional willingness, systemic reforms, and sustained engagement with the very communities whose lives are shaped by AI systems in use.

These tensions and perspective highlight the risk of treating ‘participation’ as a buzzword in both technological governance and democratic innovation, with its meaning emptied of critical content and used as a co-opting mechanism into a-democratic systems ( Lamprianou, 2013). In many AI-enabled participatory processes, algorithmic tools are used to filter, structure or even generate citizen input ( Gerdes, 2022). Yet this raises fundamental questions that critical and humanist agendas must include: who is designing these systems? Whose knowledge counts as ‘relevant’? And who is rendered invisible through these seemingly inclusive mechanisms?

Our collective discussion approached these questions through a feminist and decolonial lens, emphasising how AI systems, if left unexamined, may entrench the very exclusions they claim to solve. A particular concern is with how individuals who already face marginalisation due to their gender, class, ethnicity, geography, or bodily conditions are further excluded or distorted by AI-mediated systems. If these systems are used to inform policy, allocate resources, or shape public discourse, then the stakes of participation are existential, not merely procedural. As with the other priorities, we identified these questions and principles through sharing ideas on several areas of inquiry. For instance, claims for participation are to be critiqued in the absence of key interventions on the reproduction of sexist socio-technical relations. Think of how AI-enabled harms (such as deepfakes) are politically classified: while non-sexual deepfakes of male politicians are treated as national security threats, gendered or sexualised deepfakes are often trivialised ( Gosse & Burkell, 2020).

The third priority concerns the environmental and geopolitical implications of AI, particularly the tensions between development models, resource consumption, and global justice ( McQuillan, 2022). While the ecological costs of AI, such as water usage, energy consumption, and e-waste, are increasingly acknowledged, they remain largely disconnected from mainstream research and policy agendas. The workshop placed this paradox at the centre of the principles of critical humanist agendas: how can researchers contribute to preventing a future where the white West continues to maintain its consumption habits under the guise of ‘green AI’ ( Verdecchia *et al.*, 2023) while the ‘Global South’ is simultaneously blamed for its developmental aspirations and exploited for its resources ( Muldoon & Wu, 2023)? This priority demands both conceptual and empirical innovation. On one hand, it calls for re-theorising AI as part of a broader political economy of planetary computation. On the other hand, it demands grounded case studies on the socio-environmental consequences of AI infrastructure, from mining and supply chains to cloud data centres and satellite constellations.

What emerges from these three priorities is not a static research plan but a dynamic and pluralistic set of fresh agendas. The encounter with diverse experiences from the workshop forged a common ground for future research on AI, society and politics: one that practices collaboration and generativity to cross-fertilise diverse disciplines in arts, humanities and social sciences (at least) for a radical change of perspective.

## Our AI. Reclaiming agency through critical and humanist perspectives

Thinking about challenges, impacts, and agendas requires questioning agency, the autonomy and capacity to make a difference ( Elder-Vass, 2010). Do we, as researchers, have agency in examining how AI taps into and reshapes social relations? And at the points where agency is ‘chained’ by structured power to unequal relations, which are the traction points to unchain our liberatory potentialities? And if we consider society at large, where are the lived experiences that may work as prompters of liberation?

With this essay, we have identified two core perspectives to change how we look at AI, society and politics.

First, there is a need to be critical. This means that research is solely essential to uncover exploitation, critiquing structured power, and envisioning better forms of human organisation ( Donoghue, 2018). Especially in the field of evolving human/techno-apparatuses relations, being critical requires changing the perspective from which we look at things. While extant research on AI, society and politics has opened relevant routes of inquiry, too often the perspective is on two ‘objects’, institutions and technical systems, and on how better regulations (from the top down) and increased digital and AI literacy (from the bottom up) can reduce bias and destructive behaviours in AI usages (see, for instance: Cihon *et al.*, 2020; Mazzucato *et al.*, 2022). However, these approaches leave the core challenges we have identified throughout unchallenged. If we recognise that one strict definition of efficiency is problematic as it does not allow creativity and generativity, what is the alternative? Who would consider research that is dedicated to being anti-efficiency impactful? At the same time, when we think about participation, there is too much tendency to incorporate AI in participatory processes that do not address the key problem of how to facilitate/enable/favour the inclusion of those who are structurally excluded. While developing critical perspectives on AI, politics, and society is an open-ended and contested logic, there is a fertile ground to inform new routes of research. The constant reflection on the assumptions of how we conduct research, the search for alternatives, the constraints of different spaces of social possibilities, and the change in the points of observations are key principles for looking differently at the phenomena imbricated in the socio-technical relations we have presented throughout.

The claim for critical perspectives requires taking feminist and decolonial epistemologies seriously ( Adam, 1995; Lugones, 2008). They are not just critical contributions that the mainstream North-centric research permits, tolerates or co-opts, but are incredibly powerful heuristics for reclaiming human agency in the social relations mediated by technological systems ( Miragoli, 2025). The situatedness of who uses AI, what for, and within which institutions cannot be an appendix of research. It must become the core of a new humanist and critical perspective on these relations. This means that there is no one-size-fits-all good AI, as technological systems and those who use them are always situated in specific social and political regimes. Each regime comes with specific asymmetric power relations and intersections of exploitation that resist one ‘good AI’ for all, everywhere, every time. As our collective reflection has proposed, there is an emerging spectrum of critical positions in scholarship on society on AI that ranges, from being anti-AI, that is to say, the idea that there are whole new technologies that humans should have agency to resist and reject, to be better-AI oriented, that is the case when researchers engaging with stakeholders are normatively committed to bias reduction and the design of participatory processes to overcome digital exclusion. So far, these two sides of the spectrum do not have sufficient space for dialogue. This, instead, is essential. And taking humanist and critical perspectives is key to empowering both, without dismissing the dialectics and tensions inherent in genuinely pluralist research practices.

The essay has proposed critical and humanist perspectives to re-purpose and re-focus their ways of looking at social relations mediated by and through AI.

With this essay, we propose three core principles to this reclaiming process:

1. If a common trait of actual current AI development can be uncovered, it is that social systems whereby few techno-oligarchs control the infrastructure and narrative over technology are inherently a-democratic (at best): the current state of things must not be normalised, but counteracted.
2. If AI may serve the common good, it must be ‘us’ as humans to have the most extensive range of options and choices on what directions this may take. There must always be a switch-off button if we are to choose to keep it going and the choice of which systems for which purposes must be a collective common good.
3. Pluralism is not a decorative element of a-democratic regimes: if human creativity is put at risk by epistemological loops in AI circuits of knowledge production, enacting pluralism of experiences and perspectives must be at the core of every research process with a critical lens.

The reflections in this essay also serve as a call to further advance reflection. There may be some over-hype around the real impacts of AI. Yet, there is at least a constitutive element in how discourse on AI is permeating deeply into our lives. As such, this can provide an opportunity to become more reflexive on what it means to be researchers, and what it means to be critical in the 21st century.

## Ethics and consent

Ethical approval and consent were not required, as the essay stems from the authors' own conceptualisations.

## Data Availability

Data from the workshop, including pre-event anonymised survey responses, and notes from Lab Sessions one and two, as mentioned in the essay, are available on Zenodo Link: https://doi.org/10.5281/zenodo.17272872 ( Guglielmo, 2025), with license Data are available under the terms of the Creative Commons Attribution 4.0 International license (CC-BY 4.0).

## Footnotes

^1^ Disciplines included: politics, political economy, constitutional law, gender studies, media and arts, international relations, sociology. Participants reflected gender balance and various cultural backgrounds. Their academic affiliations are in: Germany, Belgium, Spain, United Kingdom, the Netherlands, Czech Republic.
