# Supplementary material for: Us and artificial intelligence: Questioning agency and the need for critical and humanist perspectives
Source: Open Res Eur. 2025 Oct 16;5:318. [Version 1] doi: 10.12688/openreseurope.21477.1 (PMC13560549; doi:10.12688/openreseurope.21477.1)
Supplement: Peer review history

# Reviewer report: Reviewer response for version 1

**DOI:** 10.21956/openreseurope.23231.r62812

**Published:** 2025-11-22

**Authors:** David Bailey

**Affiliations:** 1 University of Birmingham, Birmingham, England, UK

The article presents an excellent and innovative discussion of some of the key questions and challenges that researchers and practitioners face in considering how to study, understand and advocate for alternatives forms of development of, AI. It is based on a collaborative exercise stemming from a two-day workshop at the University of Valencia, in which participants were asked to engage in a series of highly innovative and extremely productive and experimental methods of collaboration, theory-building, reflection and debate, in order to bring to the surface a series of shared insights, concerns and suggestions.

 The paper does a really excellent job of introducing the reader both to the methods employed in the workshop, and the debates and observations that arose as a result of the different collaborative and reflexive exercises. In doing so, it highlights five core challenges/questions that face the use of AI in terms of the possibility for it to enhance human agency in an emancipatory or socially-valuable way. These are: the question of efficiency (for whom, and in what form), the question of participation (or exclusion), the question of what forms of knowledge are created by AI, the problem of a lack of historicity, and the problems of prediction associated with a technological development which itself is not guaranteed to be sustainable in terms of our shared future. Alongside these challenges, the authors together focus on potential principles regarding how we might think, study and do AI differently, focusing especially on the need for a critical/humanist perspective, and ending with a call for AI research to foreground three core principles: to challenge the current a-democratic form of AI, to ensure that AI is human-led in its nature, and to centre pluralism. 

 The paper overall amounts to a major and highly significant contribution to our thinking and collective process of reflection by AI-critical scholars. It brings together an extremely impressive  and highly relevant group of researchers from across a range of backgrounds. The workshop methods used and described are really outstanding in terms of methodological innovation and the findings that they have generated.

 My only suggestions are therefore really around areas that the authors might consider in terms of further clarifications for the reader, none of which I would consider essential (but nevertheless some consideration or clarification might improve the paper if the authors are so inclined), as follows:

 (1) page 7 - the authors mention the 'deleterious effects of quantified efficiency' - but perhaps some elaboration on what these deleterious effects are would be helpful.

 (2) also page 7 - the authors ask 'If AI is used to 'streamline' citizen input or 'enhance' participatory budgeting, who decides which inputs matter? Who interprets dissent?' - but this doesn't seem to be directly answered in the text. I would think that the answer is the particular LLM that is used to perform these tasks? In which case, don't we know the answer? - the LLM - and isn't the broader concern not that we don't know who will do these tasks, but rather that we know it is an LLM (and not deliberating humans) and that this is itself the problem?

 (3) The paper makes a strong case for a critical perspective and humanism. But I think some more work could have been done to spell out what exactly that means. Page 8 mentions 'situatedness, plurality, and narrative agency' with a reference to bell hooks. But some further elaboration of why these principles in particular are chosen, and in what way hooks is responsible for developing these concepts, would have been helpful. Likewise page 12 (conclusion) has some further discussion of what it means to be critical and humanist - 'to uncover exploitation, critiquing structured power, and envisioning better forms of human organisation' - but again it's not entirely clear why this is considered to be critical/humanist. Some further elaboration of what exactly it means to be critical and humanist would therefore I think have been helpful. I find Andrew Sayer's article on this question to be quite helpful and perhaps worthwhile incorporating in some way, and which I think would also fit broadly speaking with the approach that is being advocated: Sayer, A. (2009). Who’s Afraid of Critical Social Science? *Current Sociology*, *57*(6), 767-786. Related, I think there is a possibility that the article's call for a humanist approach might stand accused of essentialising what it means to be human/humanist, prompting again a need for a bit more of a detailed discussion of what it means to be 'human' and why we should privilege such a value (and indeed whether to do so is as unproblematic as the paper perhaps suggests). I think the above contribution from Sayer can also help on this question.

 (4) page 9 - the authors discuss how AI as a system might have a cannibalising effect - with reference to Nancy Fraser's book using the same concept - but I'm not sure whether Fraser is directly referring to AI when she develops this theme (I think she's more generally talking about capitalism?) - perhaps some clarification on this point?

 (5) page 10 - the authors describe what sounds like a really very interesting simulation exercise that used agentic AI chatbots 'designed with GPT-4o' - but perhaps some elaboration on how this was done would be helpful. In particular, I wasn't sure whether the chatbots *were* GPT-4o, or whether GPT-4o had designed them itself (vibe coding?). If the latter then this would also be quite interesting to the reader, I would think. Either way some elaboration on how these chatbot simulators were created would I think be of interest to the reader. This would also help later in the same section, where the authors say that these experiments 'offered critical insight into the simulation of political agency by GenAI models, how such systems are trained to emulate rhetorical strategies, project ideological stances, or manage dissent' - all of which sounds very interesting, but I think the reader would have benefited by having more information to clarify *in what way* does Gen AI do all of these things?

 6) Also in the same section, the authors describe how feminist scholars engaged with the chatbot on questions of EU foreign policy, only to find that AI systems 'can re-inscribe hierarchies of knowledge and authority' - but more could have been said I think, again, to help the reader to understand *in what way* did AI do this re-inscribing?

 7) page 11 - the discussion of scenarios that were mapped out during the workshop sounds very interesting - but the reader is not told exactly what these alternative scenarios looked like. Perhaps this could be added in?

 8) The authors mention how a feminist and decolonial lens allows us to understand how AI systems might entrench the exclusions they claim to solve. But perhaps more detail on how this entrenching happens and how we know it's existence when it occurs might be of interest. Reference to recent research by one of the co-authors on this could perhaps be used to illustrate this?: Hundt, A., Azeem, R., Mansouri, M. *et al.* LLM-Driven Robots Risk Enacting Discrimination, Violence, and Unlawful Actions. *Int J of Soc Robotics* (2025).

 9) the concluding section - the authors claim to identify 'two core perspectives' - with the first clearly labelled 'Firstly' - but I couldn't find a corresponding 'Secondly'.

 10) also in the conclusion - the third principle - 'pluralism' - I wasn't entirely sure how or why pluralism is advocated here. Is a critical and humanist approach the same as a pluralist one? I'm not sure. I would think that some interpretations of what we mean by pluralism are relativist in terms of the values that are being advocated; whereas I would think that some interpretations of critical/humanist thinking is committed to some notion of moral truths (which perhaps is not relativist?). Is this an inconsistency?

 Finally, some minor typo/grammatical issues that might be worth addressing:

 i) page 5 - 'wealthier job positions' - should be 'higher paid' or something like that, rather than 'wealthier'?

 ii) page 6 - a couple of instances of 'the AI' where I think it should be just 'AI'?

 iii) top of page 7, second column - I'm not sure what 'striking dialectics' refers to - maybe change?

 iv) the end of section "AI, politics and society" - the authors refer at the very end to 'a process in need of critique and reflexivity' - I think this isn't quite the same meaning as was developed in this section. I think (although I may have misunderstood) that a rephrasing along the following lines is closer to the argument that the authors developed: ''also requires critique and reflexivity as intrinsic elements of any research that claims to be impactful' (or something like that).

Is the argument persuasive and supported by appropriate evidence?

Yes

Is the work clearly and cogently presented?

Yes

Is the topic of the essay discussed accurately in the context of the current literature?

Yes

Does the essay contribute to the cultural, historical, social understanding of the field?

Yes

Reviewer Expertise:

political economy, social movements, forms of resistance/dissent, specifically with a recent focus on the contestation of AI as a new capitalist technology

I confirm that I have read this submission and believe that I have an appropriate level of expertise to confirm that it is of an acceptable scientific standard.

# Reviewer report: Reviewer response for version 1

**DOI:** 10.21956/openreseurope.23231.r62524

**Published:** 2025-11-14

**Authors:** Rebecca Scheffauer

**Affiliations:** 1 Freie Universitat Berlin Institut fur Soziologie (Ringgold ID: 54201), Berlin, Berlin, Germany

I appreciate the opportunity to review this interesting essay and thank the authors for highlighting several extremely relevant aspects regarding AI. However, there are some issues I would like to address. It is somewhat difficult to criticize the composition of this piece and of what is mentioned/left out as it is a reflection of what was discussed during a conference. Obviously, what did not come up then is not part of the essay. Nevertheless, there are some omissions and other problems that need to be taken care of before this could be indexing.

 My first point is probably the easiest to fix as it regards the structure of the essay. Oftentimes the authors highlight “5 key challenges”, or other short lists of points that are discussed. However, it is oftentimes difficult to find the listed ideas within the headings chosen in the rest of the essay. I do not know what the journal policy is in this regard, but numbering headings would be extremely beneficial here, as it would allow the authors to easily reference in brackets which section of the paper tackles a certain idea.

 On page 4, the authors mention that researchers often do not have the space to reflect on the consequences of AI use. I find this statement problematic, as there are plenty of papers discussing problems and limitations of AI, and even papers that investigate direct applications usually at least address some of the consequences the authors are getting at in their discussion section when they consider the implications of their findings.

 I find it interesting that the authors mention “political” questions of AI but leave out any discussion of things such as legislation regarding AI. This is a major factor in shaping what can or cannot be done with AI and how it will be done in the future. Thus, completely ignoring this area seems a glaring omission to me.

 The last paragraph in the first column on page 5/first paragraph in the second column can be deleted. The limitations should be discussed in a limitations section of the paper (which needs to be added!); the other information is not entirely relevant. Similarly, the paragraph on page 6 starting with “ultimately, the methodology” does not contribute too much to the overall narrative and needs to be removed. There already is a lot of explaining going on concerning the format under which the ideas discussed in the essay were derived, which is – to a degree – necessary. But this section needs to be a bit shorter.

 When the authors discuss efficiency of AI, a topic that is not properly addressed is “accurate vs. false” decisions reached by AI. Especially as the authors bring up the examples of AI in warfare and bureaucracy, this is highly relevant.

 While the “Efficiency or what?” section covers very broad topics, the next section regarding participation seems oddly specific in contrast. There needs to be a better connection between the two issues. Moreover, while reading said section, I kept noting that the authors never address whose data it is that is reflected in AI decisions. Which data was used to train models? E.g., if data from the web was scraped, was this only English language data, etc.? This general idea very briefly comes up much later in the next section. However, as this is an extremely important topic here as well, this needs to be mentioned (and discussed in more detail) much earlier. In general, the section regarding participation lists many, many different ideas and topics, but fails to discuss many of them in depth. A mere list of potential issues is not particularly helpful, so I would encourage the authors to either streamline this section better by removing some aspects or by keeping all ideas but discussing them better.

 On page 9, the authors mention that generative AI is not a Deus ex machina. What is not discussed properly, however, is that AI is never truly innovative, as all it does is regurgitate and recycle based on the data it was fed previously. Again, this is a vital point to the authors’ arguments and, thus, should be elaborated on.

 While I found the part of the paper that details the interactions with different chatbot personalities somewhat interesting, it is very different from the rest of the paper. So different in fact, that it does not seem to fit into this essay at all. Either the authors should integrate this better in light of their previously mentioned ideas or remove this section entirely (which might be the better option, as it, quite frankly, feels like reading about a different project).

 Finally – and this somewhat circles back to the authors never touching upon legislation – the essay never addresses or even mentions the topics of privacy and data protection, which play a giant role regarding all aspects of AI. I understand that it is difficult to add this to the essay if it simply did not come up during the conference/workshop setting, but the topic cannot be ignored.

Is the argument persuasive and supported by appropriate evidence?

Partly

Is the work clearly and cogently presented?

Partly

Is the topic of the essay discussed accurately in the context of the current literature?

Partly

Does the essay contribute to the cultural, historical, social understanding of the field?

Yes

Reviewer Expertise:

I have published several works in the field of algorithms and AI touching upon different topics from ethics to applications.

I confirm that I have read this submission and believe that I have an appropriate level of expertise to state that I do not consider it to be of an acceptable scientific standard, for reasons outlined above.
